# Genomic evidence of convergence of multidrug resistance and enhanced virulence in carbapenem- and colistin-resistant *Klebsiella pneumoniae* from Turkey

**DOI:** 10.1128/spectrum.03393-25

**Published:** 2026-03-31

**Authors:** Rayane Rafei, Issmat I. Kassem, Hasna Abbass, Anahita Ghorbani Tajani, Amine Baroudi, Jouman Hassan, Fouad Dabboussi, Metin Doğan, Monzer Hamze, Jessica E. Ericson, Steven J. Schiff, Bledar Bisha, Mehmet Özdemir, Marwan Osman

**Affiliations:** 1 Laboratoire Microbiologie, Santé et Environnement (LMSE), Doctoral School of Sciences and Technology, Faculty of Public Health, Lebanese University, Tripoli, Lebanon; 2 Department of Food Science and Technology, Center for Food Safety, University of Georgia 308511, Griffin, Georgia, USA; 3 Faculty of Agricultural and Food Sciences, American University of Beirut 11238, Beirut, Lebanon; 4 Department of Animal Science, University of Wyoming 4416, Laramie, Wyoming, USA; 5 Department of Microbiology, Necmettin Erbakan University, School of Medicine, Konya, Turkey; 6 Clinical Laboratory, Microbiology Department, Nini Hospital 237177, Tripoli, Lebanon; 7 Department of Pediatrics, Penn State College of Medicine 12310 , Hershey, Pennsylvania, USA; 8 Department of Neurosurgery, Yale University School of Medicine, New Haven, Connecticut, USA; 9 Yale Institute for Global Health 198927 , New Haven, Connecticut, USA; 10 Department of Epidemiology of Microbial Diseases, Yale University School of Public Health, New Haven, Connecticut, USA; University of Kentucky, Lexington, Kentucky, USA

**Keywords:** *Klebsiella pneumoniae*, antimicrobial resistance, carbapenemase, OXA-48, KPC-2, colistin resistance, virulome, whole-genome sequencing, Turkey

## Abstract

Multidrug-resistant (MDR) *Klebsiella pneumoniae* (*Kp*) represents a growing public health threat worldwide. We performed genomic analyses of carbapenem- and/or colistin-resistant *Kp* isolates recovered from hospitalized patients at Meram Hospital in Konya, Turkey. Forty-four isolates were identified by matrix-assisted laser desorption ionization-time-of-flight mass spectrometry, and resistance phenotypes were confirmed using broth microdilution assays. Whole-genome sequencing (Illumina) and bioinformatic analyses were used to determine sequence types (STs), antimicrobial resistance (AMR) determinants, virulence factors, and plasmid profiles. The isolates belonged to five STs: ST2096 (70.4%), ST377 (13.6%), ST101 (4.5%), ST14 (2.3%), and one novel ST (ST8972), along with two untypeable strains. Clonal group 14 (CG14), comprising ST2096, ST14, and ST8972, was predominant. Global phylogenetic analysis showed that most ST2096 isolates belonged to a clade circulating in Turkey and other countries, suggesting an international spread of these high-risk clones. No plasmid-mediated *mcr* genes were detected; however, several chromosomal mutations in *mgrB* and *pmrB* were identified. Carbapenem resistance was largely driven by *bla*_OXA-48-like_ genes, with *bla*_KPC-2_ co-detected in one isolate. Notably, 77.3% of isolates exhibited convergence of genotypes with high virulence potential and AMR, and only two isolates lacked high virulence potential. These findings highlight the rising burden of MDR *Kp* in Turkey and demonstrate an urgent need for robust One Health genomic surveillance across clinical and community settings throughout the globe. The spread of carbapenem- and colistin-resistant strains harboring enhanced virulence gene profiles in different geographical locations signals lapses in antimicrobial stewardship and infection control that can be mitigated by coordinated local and global efforts to address AMR.

**IMPORTANCE:**

The global rise of *Klebsiella pneumoniae* (*Kp*) resistant to last-resort antimicrobials represents a critical threat to public health. Despite this concern, data on the genetic drivers of resistance and virulence in Turkey remain scarce, resulting in significant gaps in local and global surveillance. This study addresses this need by providing a comprehensive genomic analysis of multidrug-resistant clinical *Kp* isolates, revealing the frequent convergence of antimicrobial resistance and genetic signatures indicative of enhanced virulence in these strains. Notably, most isolates belonged to ST2096, clustering with genomes from Turkey and other countries, highlighting the international spread of these high-risk clones. The emergence of these difficult-to-treat pathogens emphasizes the urgent need for targeted action, including sustained genomic surveillance, stronger infection prevention, and improved antimicrobial stewardship. Policymakers, clinicians, and public health stakeholders must collaborate to enhance diagnostic capacity, surveillance systems, and infection control, particularly in countries where existing gaps exacerbate the spread of multidrug-resistant pathogens with high-virulence genotypes.

## INTRODUCTION

*Klebsiella pneumoniae* (*Kp*), a well-known pathogen, has a notable capacity for acquiring resistance to last-resort antimicrobials, including carbapenems and colistin. The World Health Organization designated carbapenem-resistant *Kp* as a critical-priority pathogen for research and development of novel antimicrobial strategies (1). The emergence of hypervirulent and drug-resistant *Kp* lineages is associated with difficult-to-treat infections, prolonged hospitalizations, and high complication rates, which collectively place a substantial public health risk and economic burden on healthcare systems worldwide. Hypervirulent *Kp* (hv*Kp*) are strains equipped with a broad arsenal of virulence factors, including hypermucoviscosity and siderophore synthesis (particularly aerobactin), typically associated with severe and invasive infections. Therefore, the potential convergence of multidrug resistance and hypervirulence in *Kp* has raised concerns across the globe (2).

Several studies have reported the widespread dissemination of carbapenem- and colistin-resistant *Kp* in healthcare settings in Turkey (3–6). These resistant strains have also been widely detected in community settings, highlighting the potential for environmental transmission across the human-environment continuum (7–9). Although studies characterizing circulating clones associated with carbapenem resistance are limited in Turkey, they have revealed the predominance of specific sequence types (STs), such as ST101 and ST2096 (4, 10). Moreover, the underlying genetic mechanisms driving antimicrobial resistance (AMR) and the composition of the virulome in these isolates remain poorly understood. To address this gap, we performed in-depth genomic analyses on carbapenem- and/or colistin-resistant *Kp* isolates recovered from a hospital in Konya, Turkey. Using whole-genome sequencing (WGS), our study characterized the molecular determinants of resistance, the diversity and distribution of virulence factors, the population structure, and the associated plasmid types in carbapenem- and/or colistin-resistant *Kp* isolated from Turkey. This investigation focused exclusively on hospital isolates because (i) hospitals represent critical hubs for the transmission of multidrug-resistant (MDR) *Kp*, and (ii) hospitalized patients are particularly vulnerable to infections or colonization by this pathogen, which can lead to severe and difficult-to-treat outcomes.

## MATERIALS AND METHODS

A total of 44 non-repetitive clinical *Kp* isolates, either non-susceptible to meropenem (MIC ≥ 2 µg/mL) or resistant to colistin (MIC ≥ 4 µg/mL), were collected between March and May 2019 by the microbiology laboratory of Meram Hospital in Konya, Turkey. The patients, including 31 males and 13 females, ranged in age from 5 to 83 years (mean ± SD = 50.3 ± 23.2 years).

The before isolates were selected based on their susceptibility profiles determined during routine clinical testing using the Vitek2 system (bioMérieux, Marcy L’Etoile, France) and were recovered from various sample types, including bronchoalveolar lavage (*n* = 22), blood (*n* = 16), wound swabs (*n* = 4), and urine (*n* = 2). Identification of isolates was performed using matrix-assisted laser desorption/ionization time-of-flight mass spectrometry with the Vitek MS system (bioMérieux, Version 3.0, Marcy L’Etoile, France). Resistance to meropenem and colistin was confirmed using the broth microdilution method as described in the guidelines of the Clinical and Laboratory Standards Institute (CLSI 2025-M100). The *Escherichia coli* ATCC 7624 strain was used for quality control.

Genomic DNA was extracted from overnight cultures using the QIAamp DNA Mini Kit (Qiagen, Germantown, MD, USA) according to the manufacturer’s instructions. WGS was performed on a NovaSeq 6000 platform (Illumina, San Diego, CA, USA) to generate paired-end reads. Low-quality reads were removed, and high-quality reads were assembled as described previously (11). Draft genomes were annotated using the NCBI Prokaryotic Genome Annotation Pipeline (12). Bioinformatics analyses were conducted using default parameters with ResFinder 4.7.2, PlasmidFinder 2.1, and the multi-locus sequence types (MLST) tool available at the Center for Genomic Epidemiology (https://www.genomicepidemiology.org/) to identify AMR genes and mutations, plasmids, and STs, respectively. Insertion sequences were identified using ISFinder (https://www-is.biotoul.fr/), and the presence of the *peg-344* (putative metabolite transporter) was determined via a tblastn search using accession number BAH65947.1 against the 44 genomes, with an identity cutoff of 75% and a minimum query coverage of 90%.

The Pathogenwatch platform (https://pathogen.watch/) was additionally used to predict resistance scores, chromosomal mutations associated with colistin and quinolone resistance, virulence loci and scores, capsule locus and type, and O serotype via Kleborate (version 3.2.4). Virulence scores were classified from 0 to 5 based on the presence or absence of yersiniabactin (*ybt*), colibactin (*clb*), and aerobactin (*iuc*), following previously published criteria (13). AMR was scored from 0 to 3 based on the detection of resistance to extended-spectrum cephalosporins, carbapenems, and colistin. The convergence of increased virulence and AMR was defined as the presence of high virulence potential (virulence score ≥ 3, indicating *iuc*-positive strains) together with AMR (resistance score ≥ 1, reflecting the presence of AMR determinants, including *bla*_TEM_, *bla*_SHV_, *bla*_CTX-M_, *bla*_CMY_, *bla*_KPC_, *mgrB* mutations, and related genes).

A core genome MLST (cgMLST) tree was generated in Pathogenwatch, which included our 44 sequenced genomes and other available genomes (as of 11 August 2025) belonging to ST2096 (*n* = 252), ST377 (*n* = 38), ST101 (*n* = 252), and ST14 (*n* = 252), totaling 838 genomes (see Tables S1 and S2 at https://doi.org/10.5281/zenodo.18746733). Additionally, single-nucleotide polymorphism (SNP)-based phylogeny was inferred using Parsnp (version 2.1.4) by aligning the core genomes of the 44 study isolates with ST2096 and ST377 genomes from Pathogenwatch (see Table S3 at https://doi.org/10.5281/zenodo.18746733) using NC_016845.1 (ST11) as the reference genome. Phylogenetic trees were visualized with iTOL version 7 (https://itol.embl.de/). A SNP matrix was also built using the CSI Phylogeny 1.4 available at the Center for Genomic Epidemiology (https://cge.food.dtu.dk/services/CSIPhylogeny/) with the default parameters (minimum depth at SNP positions, 10×; minimum relative depth at SNP positions, 10%; minimum distance between SNPs, 10 bp; minimum SNP quality, 30; minimum read mapping quality, 25; and minimum *Z*-score, 1.96) (14). Isolates were considered to be clonal when the number of SNPs was equal to or lower than 10 (15).

## RESULTS

### Clonal structure and phylogenetic relationships

**Fig 1:**
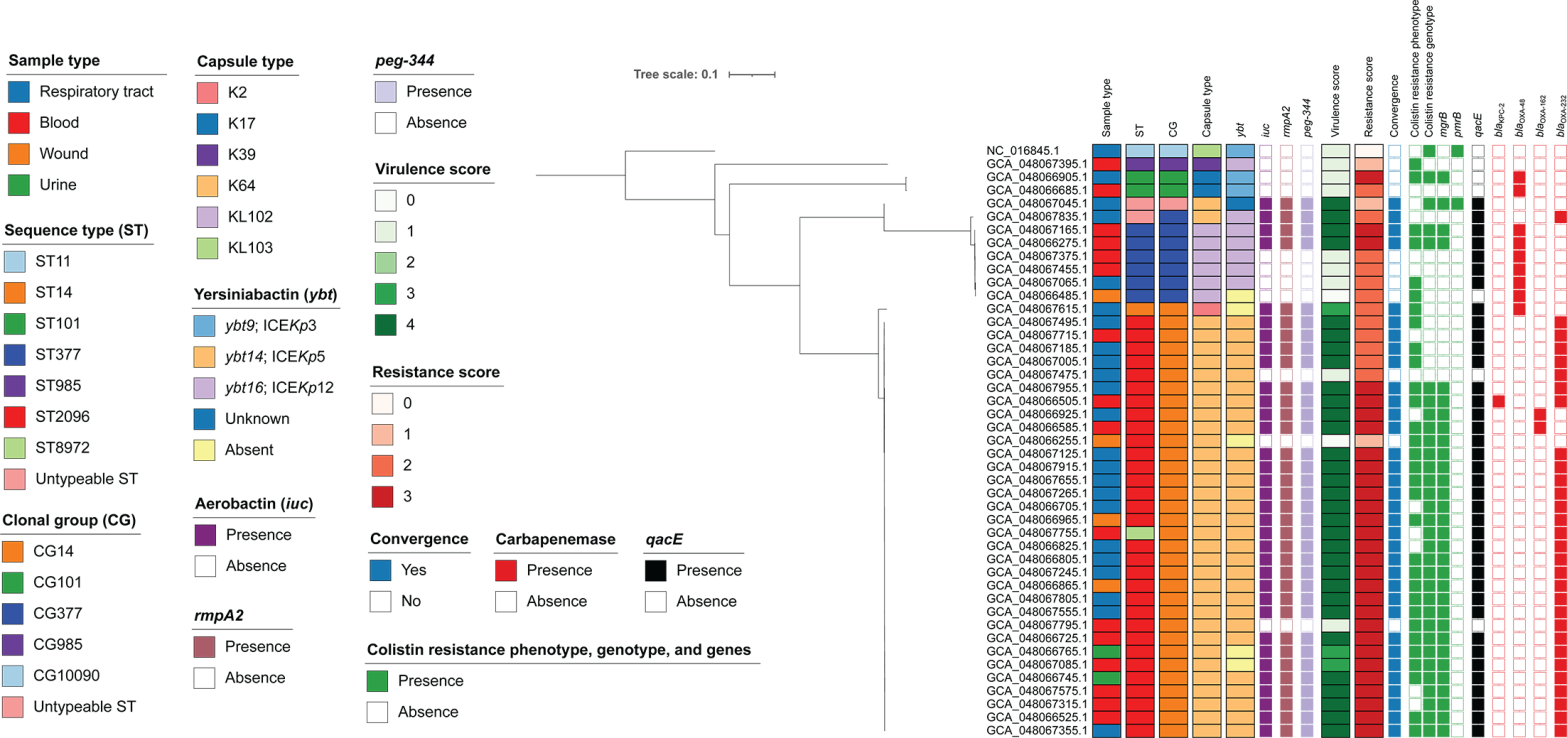
ParSNP phylogenetic tree of the 44 *Klebsiella pneumoniae* genomes analyzed in this study, rooted with the reference genome NC_016845.1 (ST11). Columns (left to right) indicate assembly accession numbers, sample origin, ST, clonal group, capsule type, yersiniabactin, aerobactin, *rmpA2*, *peg-344*, virulence score, resistance score, convergence, colistin resistance phenotype and genotype, presence of *mgrB* and *pmrB* mutations, *qacE*, and major carbapenemase genes (*bla*_KPC-2_, *bla*_OXA-48_, *bla*_OXA-162_, and *bla*_OXA-232_). The virulence score ranges from 0 to 5, where 0 = no yersiniabactin, colibactin, or aerobactin; 1, yersiniabactin only; 2, yersiniabactin and colibactin (or colibactin only); 3, aerobactin without yersiniabactin or colibactin; 4, aerobactin with yersiniabactin (no colibactin); 5, yersiniabactin, colibactin, and aerobactin. The resistance score ranges from 0 to 3, where 0, no significant resistance; 1, extended-spectrum β-lactamase; 2, carbapenemase; 3, carbapenem and colistin resistance. Convergence was defined as strains with high virulence potential (virulence score ≥ 3, representing aerobactin-containing strains) and antimicrobial resistance (resistance score ≥ 1, indicating extended-spectrum β-lactamase production, carbapenemase production, or carbapenem and colistin resistance). Isolates with intermediate susceptibility to colistin were not considered colistin-resistant.

MLST analysis showed that most of the *Kp* isolates belonged to ST2096 (31/44, 70.4%), followed by ST377 (13.6%), ST101 (4.5%), ST14 (2.3%), and one novel ST (ST8972), while two strains were untypeable (Fig. 1). These STs fell into four known clonal groups (CGs), except for the untypeable strains that could not be assigned to any CG. CG14 was the predominant group, representing 75% (33/44) of isolates, including ST2096, ST14, and ST8972. The remaining isolates belonged to CG377 (ST377), CG101 (ST101), and CG985 (ST985) (Table 1; see Table S4 at https://doi.org/10.5281/zenodo.18746733 ).

**TABLE 1:** Genomic characterization of *Klebsiella pneumoniae* isolates collected in this study, grouped according to their sequence types

Sequence type^*a*^	Number of isolates	CG^*b*^	Meropenem MIC (μg/mL) range^*c*^	Colistin MIC (μg/mL) range^*c*^	ARGs (*n*)^*d*^	*qacE^e^*	Colistin mutation (*n*)^*f*^	Plasmid types (*n*)^*g*^
ST2096	31	CG14	[2, >16]	[0.5, >16]	*bla*_OXA-1_ (30)*, bla_OXA-9_* (1), *bla_OXA-162_* (2), *bla*_OXA-232_ (28)*, bla_KPC-2_* (1), *bla_CMY-2_* (1), *bla*_CTX-M-15_ (22), *bla*_SHV-106/28_ (31)*, bla_TEM_* (2), *bla*_TEM-1A_ (20)*, aac(3)-IIa* (1), *aac(6')-Ib-cr* (30), *aadA2* (27), *aadA8b* (2), *aadA12* (1), *aph(3″)-Ib* (1), *aph(6)-Id* (1), *armA* (29), *erm(B)* (2), *oqxA* (31), *oqxB* (31), *qnrB1* (1), *mphE* (28), *msrE* (29), *tet(A)* (1), *tet(D)* (22), *tet(X)* (2), *sul1* (28), *sul2* (1), *dfrA1* (31), *dfrA12* (29), *dfrA14* (25), *catB3* (30), *floR* (1), *fosA6* (30), *fosA/fosA6* (1)	29	*mgrB*:p.Val7fs (26)	IncFIB(K) (30), IncHI1B(pNDM-MAR) (29), ColKP3 (28), IncFIB(pNDM-Mar) (24), IncI1-I(Alpha) (26), ColRNAI (1), IncC (1), IncL (2), IncFII(K) (2), IncFIB(pQil) (1)
ST377	6	CG377	[2, >16]	[0.5, 16]	*bla_OXA-1_* (6), *bla_OXA-48_* (6), *bla_CMY-2_* (6), *bla*_CTX-M-15_ (6)*, bla*_SHV-110_ (6)*, bla*_TEM-1B_ (5)*, aac(3)-Iia* (6), *aac(6')-Ib3* (4), *aac(6')-Ib-cr* (2), *aadA2* (2), *aph(3″)-Ib* (6), *aph(6)-Id* (6), *armA* (5), *oqxA* (6), *oqxB* (6), *mphE* (5), *msrE* (5), *tet(A)* (6), *tet(D)* (2), *sul1* (5), *sul2* (6), *dfrA12* (2), *dfrA14* (6), *catB3* (6), *floR* (6), *fosA* (6)	5	*mgrB*:p.Arg5fs (2)	IncHI1B(pNDM-MAR) (2), IncFIB(pNDM-Mar) (2), ColRNAI (6), IncC (6), IncFIB(K)(pCAV1099-114) (6), IncFIB(pKPHS1) (4), IncL (2)
ST101	2	CG101	≥16	[0.5, 4]	*bla_OXA-9_* (1), *bla*_OXA-48_ (2)*, bla*_CTX-M-14b_ (2)*, bla*_SHV-106/28_ (2)*, bla*_TEM_ (1)*, bla_TEM-1A_* (1), *aac(3)-Iia* (1), *aac(6')-Ib* (1), *aadA1* (2), *aadA2b* (1), *aph(3″)-Ib* (1), *aph(3')-Vib* (1), *oqxA* (2), *oqxB* (2), *mph(A)* (2), *tet(D)* (1), *sul3* (1), *dfrA14* (2), *cmlA1* (1), *floR* (1), *fosA* (2)	0	*mgrB*:p.Asp29fs (1)	IncR (2), Col440II (2), IncM1 (2), IncFIA(HI1) (1), IncFII (1), IncN4 (1), repB(R1701) (1)
Untypeable strain	2	Untypeable strain	[2, >16]	2	*bla*_OXA-1_ (2)*, bla*_OXA-48_ (1)*, bla_OXA-162_* (1), *bla*_OXA-232_ (1)*, bla*_CMY-2_ (2)*, bla*_CTX-M-15_ (2)*, bla*_SHV-110_ (2)*, aac(3)-IIa* (2), *aac(6')-Ib-cr* (1), *aadA2* (2), *aph(3″)-Ib* (2), *aph (6)-Id* (2), *armA* (2), *oqxA* (2), *oqxB* (2), *mphE* (2), *msrE* (2), *tet(A)* (2), *tet(D)* (2), *floR* (2), *sul1* (2), *sul2* (2), *dfrA1* (2), *dfrA12* (2), *dfrA14* (2), *catB3* (2), *fosA* (1), *fosA/fosA6* (1)	2	*mgrB*:p.Arg5fs (1), *pmrB*:c.C662del (1)	IncFIB(K) (2), IncHI1B(pNDM-MAR) (2), ColKP3 (2), IncFIB(pNDM-Mar) (2), IncI1-I(Alpha) (2), ColRNAI (2), IncC (2), IncFIB(K)(pCAV1099-114) (2), IncFIB(pKPHS1) (2)
ST14	1	CG14	8	4	*bla*_OXA-1_ *, bla*_OXA-48_ *, bla*_CTX-M-15_ *, bla*_SHV_ *, bla*_TEM-1A_ *, aac (3)-IId, aac(6')-Ib-cr, aadA2, armA, oqxA, oqxB, mphE, msrE, tet(D), sul1, dfrA1, dfrA12, dfrA14, catA1, catB3, fosA6*	1	–^*h*^	IncFIB(K), IncHI1B(pNDM-MAR), IncFIB(pNDM-Mar), IncL, IncFII(K), IncR
ST985	1	CG985	1	4	*bla*_CMY_ *, bla*_CTX-M-15_ *, bla*_SHV-187_ *, bla*_TEM_ *, aac(6')-Ib3, oqxA, oqxB, qnrB1, tet(A), dfrA14, fosA6*	0	–	IncFIB(K), ColRNAI, IncFII(K)
ST8972	1	CG14	4	2	*bla*_OXA-1_ *, bla*_OXA-232_ *, bla*_CTX-M-15_ *, bla*_SHV-106/28_ *, bla*_TEM-1A_ *, aac(6')-Ib-cr, aadA2, armA, oqxA, oqxB, mphE, msrE, tet(D), sul1, dfrA1, dfrA12, dfrA14, catB3, fosA6*	1	*mgrB*:p.Val7fs	IncFIB(K), IncHI1B(pNDM-MAR), ColKP3, IncFIB(pNDM-Mar), IncI1-I(Alpha)

^*a*^ Sequence type as determined by MLST.

^*b*^ CG for clonal group.

^*c*^ MIC, minimum inhibitory concentration; resistance to meropenem and colistin was assessed using the broth microdilution method as described in the guidelines of the Clinical and Laboratory Standards Institute (CLSI 2025-M100). Meropenem clinical breakpoints: susceptible ≤ 1 µg/mL, intermediate = 2 µg/mL, resistant ≥ 4 µg/mL. Colistin clinical breakpoints: intermediate ≤ 2 µg/mL, resistant ≥ 4 µg/mL.

^*d*^ ARGs found in the *Klebsiella pneumoniae* genomes, either intrinsic or acquired.

^*e*^ The number of isolates carrying the *qacE* gene.

^*f*^ Chromosome-mediated mutations potentially responsible for colistin resistance.

^*g*^ The number in parentheses indicates the number of isolates harboring each plasmid type.

^*h*^ –, no mutations associated with colistin resistance were detected.

**Fig 2:**
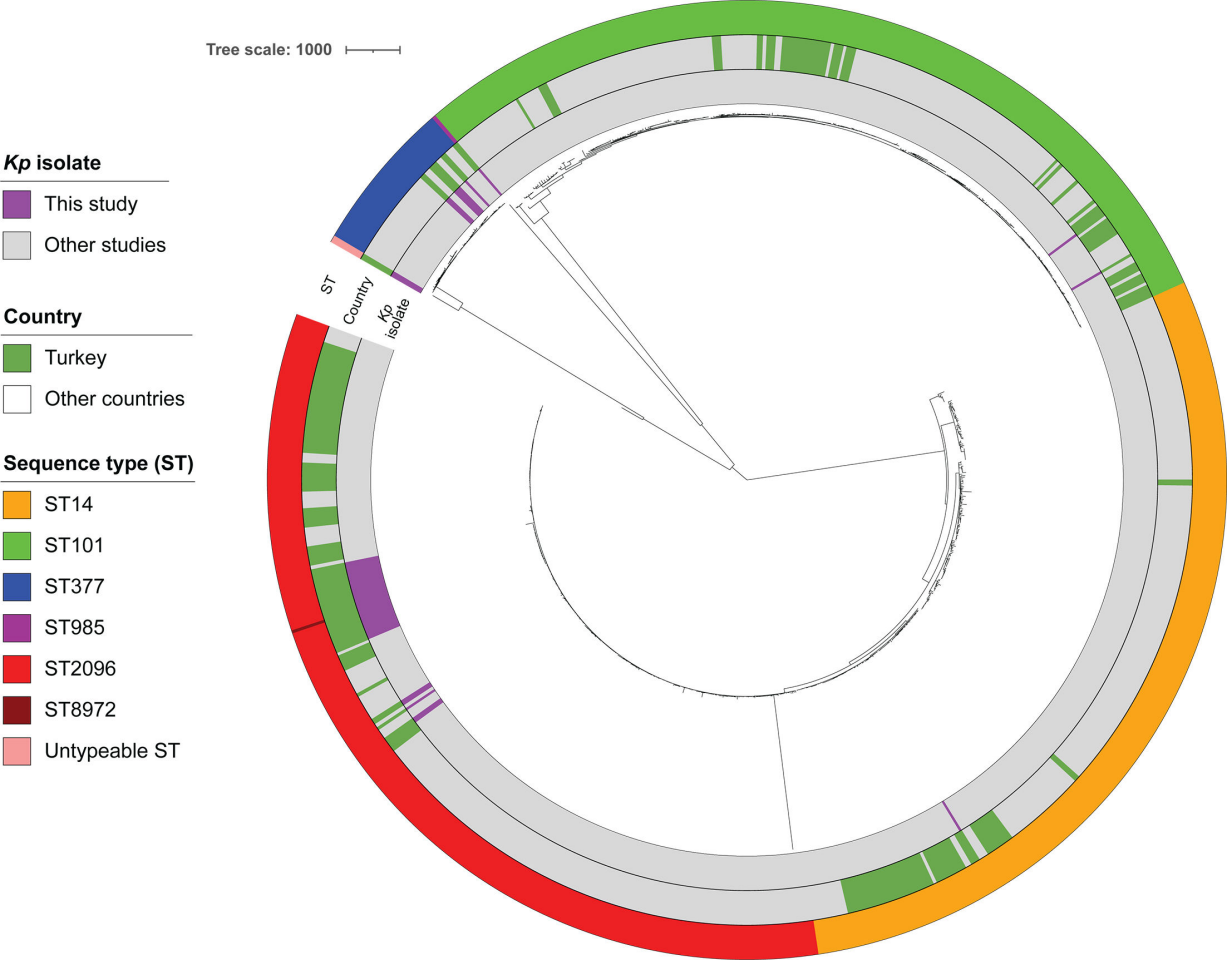
Core genome MLST phylogenetic tree of *Klebsiella pneumoniae* isolates, including 44 genomes from this study and 794 publicly available genomes (ST2096, *n* = 252; ST377, *n* = 38; ST101, *n* = 252; and ST14, *n* = 252). The innermost ring denotes study isolates, the second ring indicates genomes originating from Turkey, and the third ring represents sequence types.

**Fig 3:**
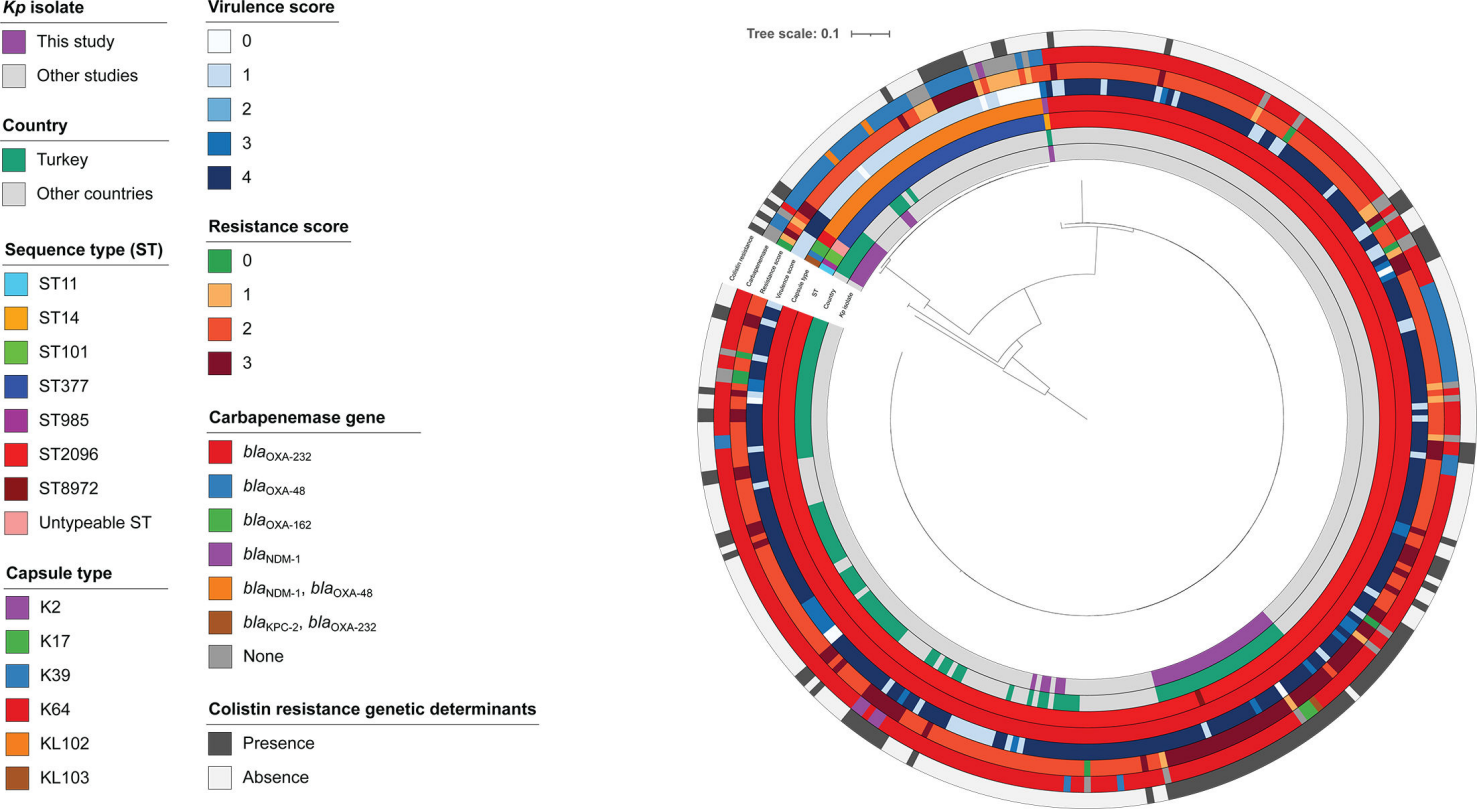
SNP-based phylogenetic analysis of *Klebsiella pneumoniae* isolates generated by Parsnp, including 44 genomes from this study and publicly available ST2096 (*n* = 252), and ST377 (*n* = 38). The tree was rooted on the reference ST11 (NC_016845.1) genomes from Pathogenwatch. Concentric circles indicate isolates sequenced in this study in the innermost ring, followed outward by isolates originating from Turkey, ST, capsule type, virulence score, resistance score, carbapenemase genes, and presence of colistin resistance determinants. Isolates with intermediate susceptibility to colistin were not considered colistin-resistant. The virulence score ranges from 0 to 5, where 0, no yersiniabactin, colibactin, or aerobactin; 1, yersiniabactin only; 2, yersiniabactin and colibactin (or colibactin only); 3, aerobactin without yersiniabactin or colibactin; 4, aerobactin with yersiniabactin (no colibactin); 5, yersiniabactin, colibactin, and aerobactin. The resistance score ranges from 0 to 3, where 0, no significant resistance; 1, extended-spectrum β-lactamase; 2, carbapenemase; 3, carbapenem and colistin resistance.

To further investigate the phylogenetic relationships of *Kp* isolates from this study alongside publicly available genomes in Pathogenwatch, we constructed phylogenetic trees for the five STs identified in our cohort (ST2096, ST377, ST101, ST8972, and ST14). In both the cgMLST (Fig. 2) and SNP-based phylogenetic trees (Fig. 3), isolates of ST2096 and ST8972 from this study clustered with other ST2096 genomes available on Pathogenwatch. ST14, although belonging to the same CG14 group, formed a distinct clade from ST2096. A similar pattern was observed for ST377, where our isolates clustered with publicly available ST377 genomes. Within our data set, SNP distances ranged from 5 to 162 for ST2096 and ST8972, 15 to 87 for ST377, and 118 for ST101. Utilizing a SNP distance threshold of 10 SNPs, three discrete genetic clusters within ST2096 could be delineated, wherein each cluster exhibited intra-group genetic diversity of approximately 10 SNPs or less (see Fig. S1 at https://doi.org/10.5281/zenodo.18746733). The predominant cluster comprised nine isolates, consisting of eight ST2096 isolates and one ST8972 isolate, while the remaining two clusters consisted of two and three isolates, respectively.

Capsular typing showed strong concordance with ST assignments. K64 was the predominant capsular type (77.3%), consistently detected in all ST2096 isolates as well as in the novel ST8972 isolate. KL102 was the second most frequent locus (13.6%), restricted to ST377, while less common types included K17 in ST101 (4.5%), K2 in ST14 (2.3%), and K39 in ST985 (2.3%). Genomic analysis confirmed that ST2096 and ST377 were consistently associated with K64 and KL102, respectively, regardless of source or country of isolation.

The most prevalent predicted O-polysaccharide serotype was O1 (86.4% of isolates). This included ST2096 (subtypes O1αβ,2α and O1αβ,2β), ST101 (O1αβ,2α), ST985 (O1αβ,2β), ST14 (O1αβ,2α), ST8972 (O1αβ,2α) and the two untypeable strains (O1αβ,2β). The remaining isolates (13.6%) had the O2 serotype, observed in all ST377 isolates (subtype O2β) (see Table S5 at https://doi.org/10.5281/zenodo.18746733).

### Phenotypic resistance to carbapenem and colistin and distribution of antimicrobial resistance genes

Based on CLSI breakpoints, dual resistance to both carbapenem and colistin was observed in 21 of the 44 analyzed isolates (47.7%; see Table S4 at https://doi.org/10.5281/zenodo.18746733). Additionally, 10 isolates were resistant to colistin but exhibited either intermediate resistance (9/10) or susceptibility (1/10) to meropenem. Conversely, 10 isolates were resistant to meropenem but showed intermediate resistance to colistin. Finally, three isolates displayed intermediate resistance to both colistin and meropenem.

The core resistance genes *bla*_SHV_, *fosA*, and *oqxA/B* were detected in all isolates, with *bla*_SHV-106/28_ and *fosA6* representing the predominant alleles. The number of acquired AMR genes per isolate ranged from 5 to 23 (median: 15) (see Table S5 at https://doi.org/10.5281/zenodo.18746733). The antiseptic-resistance gene *qacE*, associated with resistance to quaternary ammonium compounds, was present in 86.4% of genomes. Resistance scores were most frequently high (score 3), reflecting the co-occurrence of carbapenemase and colistin resistance (65.9%), followed by score 2, indicating carbapenemase without colistin resistance (27.3%), and score 1, corresponding to extended-spectrum β-lactamase production without carbapenemase (6.8%). Although plasmid-mediated *mcr* genes were absent, multiple chromosomal mutations were identified in *mgrB* and *pmrB*. Three *mgrB* frameshift variants were detected: Val7fs (*n* = 27, including 21 colistin-resistant and 6 colistin-intermediate isolates), Asp29fs (1 colistin-resistant isolate), and Arg5fs (*n* = 3, including 2 colistin-resistant and 1 colistin-intermediate isolates). A single *pmrB* mutation (c.C662del) was observed in one intermediate isolate but was not associated with colistin resistance.

Among the 43 carbapenem-non-susceptible isolates showing resistance or intermediate resistance, 38 carried *bla*_OXA-48-like_ carbapenemase genes, specifically *bla*_OXA-232_ (*n* = 30), *bla*_OXA-48_ (*n* = 10), and *bla*_OXA-162_ (*n* = 2). One isolate carrying *bla*_OXA-232_ also harbored *bla*_KPC-2_. Two carbapenem-intermediate isolates lacked carbapenemase genes but carried *bla*_CMY-2_ and/or *bla*_CTX-M-15_ in combination with mutations in *ompK36* (ompK36:c.G547del and ompK36:p.134_135insGlyAsp). Frameshift and deletion mutations in porins were also observed, including *ompK35* (i.e., Ile61fs, 4.9%; Thr315fs, 4.9%; and Tyr173fs, 2.4%) and *ompK36* (i.e., G188del, 2.4%; G547del, 2.4%; and G97del, 2.4%), which are predicted to result in porin loss or misfolding. All such porin-disrupting mutations were detected in carbapenem-non-susceptible isolates, including those with resistant or intermediate phenotypes, and all but one of these isolates also harbored a carbapenemase-encoding gene. Additional acquired β-lactamase genes included *bla*_TEM-1A_ (23/44, 52.3%), *bla*_TEM-1B_ (5/44, 11.4%), *bla*_OXA-1_ (40/44, 90.9%), *bla*_OXA-9_ (2/44, 4.5%), *bla*_CMY-2_ (9/44, 20.5%), *bla*_CTX-M-14b_ (2/44, 4.5%), and *bla*_CTX-M-15_ (33/44, 75%).

All isolates carried between one and six aminoglycoside resistance genes, most commonly *armA* (86.4%), *aac(6′)-Ib-cr* (79.5%), and *aadA2* (75%). High-level quinolone resistance was mediated by both chromosomal mutations (Asp87Gly [75%] and Ser83Tyr [95%] in *gyrA*, and Ser80Ile [95%] in *parC*) and acquired AMR genes [*aac(6′)-Ib-cr* (79.5%) and *qnrB1* (4.5%)]. All isolates carried at least one *dfrA* gene conferring trimethoprim resistance. Additional resistance determinants included genes conferring resistance to amphenicols (93%, mainly *catB3*), macrolides (91%, mainly *msrE* and *mphE*), sulfonamides (88.6%, mainly *sul1*), and tetracyclines [77%, mainly *tet(D*)] (see Table S6 at https://doi.org/10.5281/zenodo.18746733). Collectively, the acquired AMR determinants reflected 21 distinct resistance profiles (see Table S7 at https://doi.org/10.5281/zenodo.18746733).

### Distribution of virulence-encoding genes

Except for two isolates, all *Kp* exhibited a virulence score ≥ 1. In total, 8 isolates carried only *ybt* (score 1), 3 carried *iuc* without *ybt* (score 3), and 31 carried both *iuc* and *ybt* (score 4) (see Table S5 at https://doi.org/10.5281/zenodo.18746733). None harbored *clb*, salmochelin, or *rmpADC* loci. Four *ybt* lineages associated with ICE*Kp* (*Kp* integrative conjugative element) were identified, including *ybt14*/ICE*Kp*12 (*n* = 29), *ybt16*/ICE*Kp*12 (*n* = 7, including one truncated ICE), *ybt9*/ICE*Kp*3 (*n* = 2), and one untypeable variant. Thirty-four isolates carried *iuc1* and *rmpA2_8*. Convergence of enhanced virulence and AMR (virulence score ≥ 3 plus resistance score ≥ 1) occurred in 34 isolates. The *peg-344* gene was consistently present in all genomes with a virulence score greater than 3. ST2096 isolates exhibited higher virulence scores (mean ± SD = 3.61 ± 1.02) than ST377 (mean ± SD = 1.83 ± 1.72) (Fig. 3).

### Characterization of mobile genetic elements and resistance genetic backgrounds

A total of 19 different plasmids were identified in the sequenced isolates, resulting in 18 unique plasmid profiles (see Tables S8 and S9 at https://doi.org/10.5281/zenodo.18746733). Each isolate carried between two and nine plasmids (median: 5). The most prevalent plasmids were IncFIB(K) and IncHI1B(pNDM-MAR) (79.5% of isolates), ColKP3 (70.5%), IncFIB(pNDM-MAR) (68.2%), and IncI1-I(Alpha) (65.9%). Some plasmids or plasmid profiles were restricted to specific STs. For example, Col440II and IncM1 were found only in ST101, whereas the combination IncFIB(K), IncHI1B(pNDM-MAR), ColKP3, IncFIB(pNDM-MAR), and IncI1-I(Alpha) was unique to ST2096 and ST8972.

Although short-read sequencing limits the complete resolution of plasmid structures and the genetic context of AMR genes, several common arrangements or structural signatures were identified. ColKP3 carried *bla*_OXA-232_ (*n* = 30), *bla*_CTX-M-15_ (*n* = 12), and *bla*_TEM-1A_ (*n* = 7), while *bla*_KPC-2_ was located on an IncFII(K) plasmid. All *bla*_OXA-48_ genes and the variant *bla*_OXA-162_ were located within a potential Tn*1999.1*, and *bla*_CTX-M-15_ was frequently associated with IS*Ecp1*. The plasmid-borne *bla*_KPC-2_ was flanked by its characteristic insertion sequences, IS*kpn6* and IS*kpn27* (see Fig. S2 t0 S6 and Table S10 at https://doi.org/10.5281/zenodo.18746733).

## DISCUSSION

The emergence of hv*Kp* strains resistant to last-resort agents, such as carbapenems and colistin, represents a critical threat to global public health. In our study, while nearly half of isolates exhibited phenotypic resistance to both meropenem and colistin, we genomically confirmed dual resistance to carbapenems and colistin in two-thirds of isolates. This finding highlights the alarming extent of resistance and the scarcity of effective therapeutic options. Consistent with previous reports from Turkey, *bla*_OXA-48-like_ was the predominant carbapenemase among carbapenem-resistant *Kp*. It was frequently co-harbored with plasmid-encoded cephalosporinases (*bla*_CMY-2_) and/or extended-spectrum β-lactamase (*bla*_CTX-M-14b_ and *bla*_CTX-M-15_) genes. Within this group, *bla*_OXA-232_ was the most common *bla*_OXA-48-like_ variant and was primarily carried on the ColKP3 plasmid, aligning with previous findings (16). While Turkey is considered endemic for *bla*_OXA-48-like_ (17), we also detected *bla*_KPC-2_-producing *Kp*. Previous reports in Turkey have described *bla*_KPC-2_ in ST11 (18), ST147 (19), and ST258 (17); however, to our knowledge, this is the first report of its occurrence in ST2096 (see Table S11 at https://doi.org/10.5281/zenodo.18746733). The emergence of *bla*_KPC-2_ in this high-risk clone suggests ongoing diversification of carbapenemase reservoirs in the region and highlights the potential for further dissemination of resistance determinants across successful lineages. Regarding colistin resistance, although previous studies have reported *mcr-1* in *Kp* isolates from Turkey, we did not identify plasmid-mediated *mcr* genes in our collection (3). Instead, resistance was commonly associated with chromosomal alterations in the *mgrB* gene, as reported in previous studies (20). The predominant genetic change was a frameshift mutation (*mgrB*:p.Val7fs), which is predicted to abolish MgrB function, leading to constitutive activation of the PhoP-PhoQ two-component regulatory system and subsequent modifications of the lipopolysaccharide target of polymyxins (21). While these predictions align with well-established resistance mechanisms, functional studies are needed to confirm the role of *mgrB* disruptions in colistin resistance (22). Notably, *mgrB* mutations can also be found in isolates showing intermediate resistance to colistin, which are currently considered susceptible to higher colistin dosages. This highlights the complex relationship between genotype and phenotype, as resistance-associated mutations can also be present in isolates that are not phenotypically resistant (23). The *qacE* gene was commonly detected, which might further facilitate bacterial persistence in hospital environments that experience frequent use of disinfectants. The *qacE* gene might provide an additional selection advantage under biocide pressure and enhance the ability of these strains to survive, disseminate, and fuel healthcare-associated infections and outbreaks (24).

Convergent *Kp* strains combining multidrug resistance and genetic signatures indicative of enhanced virulence potential were prevalent in this cohort. They pose a significant challenge for infection control and raise serious concerns about the dissemination of high-risk clones within healthcare facilities and into the community (25). These “dual-risk” clones, harboring traits of resistance and virulence simultaneously, represent particularly dangerous pathogens as they unite clinically advantageous “high-value” resistance determinants with virulence factors that facilitate invasive disease, thereby exacerbating the AMR crisis. The carriage of two capsular polysaccharide regulator genes (*rmpA* and *rmpA2*) together with iron-acquisition systems such as siderophore gene clusters (*iuc* and *ybt*) is thought to drive the hypermucoviscous phenotype characteristic of hv*Kp* (26). In our study, these virulence determinants were widely observed, as 70% of isolates displayed a virulence score greater than 4, reflecting the co-presence of *iuc* and *ybt* genes. Although all isolates with a virulence score ≥ 3 carried truncated forms of *rmpA2*, which may reduce or abolish the expression of the classical hypermucoviscous phenotype, their virulence potential remained high. This was likely due to compensatory virulence factors such as *peg-344*, a putative transporter gene and one of the five molecular biomarkers of hv*Kp* (27). While we have not demonstrated the presence of all classical hv*Kp* biomarkers, these isolates nevertheless carried a broad repertoire of virulence genes. These findings underscore the multifactorial nature of *Kp* pathogenicity, where the loss of function in one determinant can be offset by the presence of others, ensuring persistence of high virulence. Capsular serotyping further reinforced this conclusion; the K64 serotype predominated in our cohort and is recognized as one of the most common MDR-hv*Kp* lineages, frequently associated with community-acquired invasive infections in Southeast Asia (28).

MLST analysis revealed multiple genetic backgrounds among the *Kp* isolates, with ST2096 being predominant. SNP analysis further identified homogeneous subgroups within ST2096 with 10 SNPs or fewer, suggesting the potential presence of cryptic transmission chains and spread of nosocomial clones that may represent an undetected outbreak. Although the absence of detailed clinical data limits our ability to establish transmission linkages, these findings underscore the value of genomic surveillance in detecting concealed outbreaks and guiding infection control strategies. Genomic comparisons showed that nearly all ST2096 isolates in this study formed a highly homogeneous group, clustering within a clade that also included isolates from Turkey and other countries (Fig. 2 and 3). This finding highlights the broad geographic dissemination of this high-risk lineage. ST2096 has recently emerged as an MDR and hypervirulent clone, implicated in several outbreaks across the Middle East (29, 30). Integrating our ST2096 data set (*n* = 31 genomes) with 252 publicly available ST2096 genomes in Pathogenwatch further illustrates the dual-risk nature of this lineage, as 77% of isolates carried a virulence score of 4, and 26% exhibited combined resistance to carbapenems and colistin (resistance score = 3). Consistent with previous reports indicating that 63% of ST2096 isolates worldwide are hv*Kp* (31), our findings confirm the convergence of potential hypervirulence traits and extensive drug resistance in ST2096, reinforcing its capacity to cause severe, hard-to-treat infections.

ST377 was also detected in our cohort, with one-third of isolates exhibiting combined carbapenem and colistin resistance, consistent with reports from Iran describing this lineage as a highly resistant clone associated with clinical infections (32). While the proportion of ST377 isolates resistant to both carbapenems and colistin was comparable to ST2096, genetic signatures indicative of enhanced virulence were markedly less frequent, with only 4.5% of isolates reaching a virulence score of 4. This suggests that, unlike ST2096, the threat posed by ST377 is primarily resistance-driven rather than the result of a convergence between resistance and virulence, shedding light on the diverse evolutionary trajectories shaping high-risk *Kp* lineages and setting the stage for novel nationwide large-scale studies to investigate *Kp* circulating clones and their association with virulence and drug resistance at the human-animal-environment interface.

While short-read sequencing limits the resolution of AMR gene contexts and plasmid architectures, our analysis revealed potential similarities in the genetic environments and associated mobile genetic elements carrying these resistance genes, consistent with previous reports (33). These findings suggest that the spread of AMR in this data set is likely driven by horizontal gene transfer mediated by mobile genetic elements rather than by clonal expansion; however, confirmation of plasmid structures and transmission dynamics would require long-read sequencing (34, 35). The detection of the large conjugative IncFII(K) plasmid as a vector for *bla*_KPC-2_ aligns with previous global data (36, 37) and highlights a different pattern for KPC-2 spread, which is driven by plasmid conjugation. Although a non-conjugative plasmid, the *bla*_OXA-232_-harboring ColKP3 plasmid identified here, primarily in ST2096 as well as in different STs worldwide, was recently detected in outer membrane vesicles, potentially facilitating the horizontal spread of *bla*_OXA-232_ among Enterobacterales (38). This also suggests that plasmids with restricted horizontal transfer potential can be major drivers of resistance persistence and dissemination in a hospital setting. Beyond plasmid backbones, the localization of resistance genes within highly mobile genetic structures, such as Tn*1999.1*-like transposon, IS*Ecp1*, IS*kpn6,* and IS*kpn27*, provides further insights into their mobilization potential and the intricacy of such structures (16, 39–41).

To our knowledge, this study represents the first comprehensive genomic analysis in Turkey examining the epidemiology of *Kp* isolates exhibiting phenotypic resistance to both carbapenems and colistin. However, we acknowledge several important limitations. The epidemiological inferences drawn from this study are based on isolates collected from a single center and therefore may not reflect the national epidemiology of carbapenem- and colistin-resistant *Kp* in Turkey. Moreover, as this analysis focused on a selected subset of isolates exhibiting resistance to carbapenems and/or colistin, it may not capture the overall local epidemiology of *Kp* within the hospital. Additionally, our conclusions about the plasmidome and associations of certain plasmids or mobile elements with resistance genes were extrapolated based on short-read sequencing, which is not optimal for resolving plasmid structures. The absence of detailed clinical data, including patient outcomes and length of stay, limits our ability to assess potential associations between ST, virulence score, and clinical outcomes. Future studies incorporating comprehensive clinical metadata will be essential to determine whether these convergent clones represent a direct and clinically significant threat to patient health. Moreover, convergence was inferred from genomic signatures combining AMR and virulence, enabling the identification of genotypes with both multidrug resistance and high virulence potential. While these genomic indicators provide strong evidence of convergent lineages, phenotypic validation, such as assessment of hypermucoviscosity, was not performed and could provide additional confirmation in future studies. In conclusion, our findings highlight *Kp* ST2096 as the predominant high-risk clone in Turkey, combining carbapenem and colistin resistance with genetic signatures indicative of enhanced virulence. The emergence of these convergent lineages underscores the urgent need to strengthen One Health surveillance, enforce effective infection control, and advance evidence-based antimicrobial stewardship. If we fail to address this challenge effectively, dual-risk *Kp* lineages like ST2096 will further erode treatment options and exacerbate the global AMR crisis.

## Data Availability

All 44 genomes were available under the BioProject number PRJNA1223660 in NCBI.
